# Transcriptomic Analysis of LNCaP Tumor Xenograft to Elucidate the Components and Mechanisms Contributed by Tumor Environment as Targets for Dietary Prostate Cancer Prevention Studies

**DOI:** 10.3390/nu13031000

**Published:** 2021-03-19

**Authors:** Lu Yu, Robert W. Li, Haiqiu Huang, Quynhchi Pham, Liangli Yu, Thomas T. Y. Wang

**Affiliations:** 1 Department of Nutrition and Food Science, University of Maryland, College Park, MD 20742, USA; yulu0640514@gmail.com (L.Y.); tennisqiu@gmail.com (H.H.); lyu5@umd.edu (L.Y.); 2 Animal Parasitic Diseases Laboratory, Beltsville Area Research Center, ARS, USDA, Beltsville, MD 20705, USA; robert.li@usda.gov; 3 Diet, Genomics and Immunology Laboratory, Beltsville Human Nutrition Research Center, ARS, USDA, Beltsville, MD 20705, USA; quynhchi.pham@usda.gov

**Keywords:** diet, prevention, prostate cancer, transcriptomic analysis, tumor xenograft, RNA-seq

## Abstract

LNCaP athymic xenograft model has been widely used to allow researchers to examine the effects and mechanisms of experimental treatments such as diet and diet-derived cancer preventive and therapeutic compounds on prostate cancer. However, the biological characteristics of human LNCaP cells before/after implanting in athymic mouse and its relevance to clinical human prostate outcomes remain unclear and may dictate interpretation of biological efficacies/mechanisms of diet/diet-derived experimental treatments. In this study, transcriptome profiles and pathways of human prostate LNCaP cells before (in vitro) and after (in vivo) implanting into xenograft mouse were compared using RNA-sequencing technology (RNA-seq) followed by bioinformatic analysis. A shift from androgen-responsive to androgen nonresponsive status was observed when comparing LNCaP xenograft tumor to culture cells. Androgen receptor and aryl-hydrocarbon pathway were found to be inhibited and interleukin-1 (IL-1) mediated pathways contributed to these changes. Coupled with in vitro experiments modeling for androgen exposure, cell-matrix interaction, inflammation, and hypoxia, we identified specific mechanisms that may contribute to the observed changes in genes and pathways. Our results provide critical baseline transcriptomic information for a tumor xenograft model and the tumor environments that might be associated with regulating the progression of the xenograft tumor, which may influence interpretation of diet/diet-derived experimental treatments.

## 1. Introduction

Understanding the biology of prostate cancer is critical for developing successful diet/diet-derived compound preventive and therapeutic strategies for this disease. To this end, rodent models are often used to simulate human prostate cancer initiation and development processes and to test diet/diet-derived compound prevention and treatment modalities [1]. Among the numerous rodent prostate cancer models, genetically engineered mouse (GEM) [2], chemical-induced carcinogenesis [3], and tumor xenograft models [4] are extensively used to investigate the molecular and cellular mechanisms underlying prostate cancer progression [5,6]. There are several GEM models which include transgenic adenocarcinoma of the mouse prostate (TRAMP), LADY, PTEN knockout, and c-Myc overexpression mice models [1,7,8]. The chemical-induced carcinogenesis model involves the use of chemical carcinogens, or in combination with testosterone, to induce prostate carcinomas in the hosts. Xenograft models, compared to GEM and chemical carcinogenic models, have the advantage of allowing researchers to introduce known human prostate cancer cells into the hosts, which is thought to be more relevant to the study of human prostate pathophysiology than the other two models [7]. However, the available prostate cancer models are not perfect and do not allow one to address specific questions [9,10].

BALB/c Nu/Nu, NMRI/Nu, Severely Combined Immune Deficient (SCID), and RAG and NOD/SCID are the four major hosts used in prostate xenograft study [11]. The BALB/c Nu/Nu model has a deficient cell-mediated immune response by lacking a functional thymus due to Foxn1 mutation [12]. BALB/c Nu/Nu mouse is one of the most common hosts for xenotransplantation of human tumors allowing tumor size measurement [13]. Xenograft can be established by implanting patient-derived tumor tissue [14], orthotopic injection of cultured human cell line into mouse prostate [15], or subcutaneous injection of cultured human cell line into immunodeficient mouse [5,6,16]. The patient-derived tumor model requires patient subjects, thus, introducing individual variation [17], which is difficult to replicate the study. Drawbacks of an orthotopic model are that the transplantation requires expertise and the tumor growth in mouse prostate would be difficult to monitor over a period of time [15]. A subcutaneous xenograft model, while limited to a particular cell line, has the advantages of increased feasibility of the experiments, allows the measurement of tumor sizes, and lowers the difficulties of replicating the study. The most commonly used cell lines for prostate xenograft models are human prostate cancer LNCaP (androgen sensitive), DU145, and PC-3 (androgen nonsensitive) cell lines [1,17]. Both PC-3 and DU145 are representatives of the early androgen depletion independent prostate cancers [18], and the LNCaP cells are hormonally responsive from a metastatic lesion of human prostatic adenocarcinoma [19]. The LNCaP cell line is usually used to study the regulation of prostate cancer progression in response to hormones [20].

Compared to cells in culture, the in vivo xenograft tumor can allow studies of more complicated interactions, including tumor microenvironment [21], exposure to circulating androgen [22], blood flow [23], hypoxia [24], interaction between implanted human cells and mouse immune cells [25], as well as body metabolism [26]. These interactions may lead to altered gene expression and regulation of the development and progression of implanted human prostate cancer cell/tumor. However, precise information on tumor microenvironment and the impact on tumor cells remain unclear and warrants further elucidation. In addition, the main purpose of animal study is to mimic or estimate the clinical cancer progression, study the mechanisms, and provide preventive or therapeutic strategies. The relevance between the mouse xenograft model and clinical human prostate tumor studies is critical, but it is not well documented in prostate cancer research.

The main goal of this study is to provide basic molecular information of the xenograft in vivo platform to facilitate targets/mechanisms identification from dietary prostate cancer preventive studies. To further elucidate the interaction in the tumor microenvironment and what information regarding cancer progression stem from a xenograft model, this study utilized next generation sequencing (NGS) technology [27] to compare the global transcriptomes of human prostate cancer LNCaP cells before and after implanting in the athymic BALB/c Nu/Nu mouse xenograft model. Coupling with in vitro system to model androgen exposure, cell–matrix interaction, inflammation, and hypoxia, results from this study seek to provide specific information on how the prostate tumor cell xenograft model progresses in the carcinogenesis process, possible mechanisms involved, and potential targets for diet/diet-derived compounds preventive studies.

## 2. Materials and Methods

### 2.1. Chemicals and Reagents

Cell culture medium RPMI-1640 medium with or without phenol red, fetal bovine serum, and TRIzol reagent were purchased from Life Technologies (Grand Island, NY, USA). Antibiotic agent 100X penicillin–streptomycin mix (pen–strep) was obtained from Sigma-Aldrich (St. Louis, MO, USA). Matrigel was obtained from BD Biosciences (Mansfield, MA, USA). TaqMan Fast Universal PCR Master Mix, primers were from Life Technologies (Grand Island, NY, USA). All chemicals were analytical reagent grade.

### 2.2. Cell and Cell Culture

The LNCaP human prostate cancer cell line was purchased from American Type Culture Collection (ATCC, Manassas, VA, USA). Cells were cultured in RPMI 1640 medium with phenol red containing 10% fetal bovine serum (FBS) and 1% pen–strep at a concentration of 2.5 × 10^5^ cells/mL in 175 cm^2^ flasks at 37 °C with 5% CO_2_.

### 2.3. In Vivo Xenograft Bioassay

The experimental protocol (#12-030) was approved by the USDA Beltsville Area Animal Care and Use Committee. Male athymic nude mice (BALB/c nu/nu, 20–22 g, 5–6 weeks old; Charles River, Frederick, MD, USA) were individually kept in HEPA filter-top cages, and they consumed food and fresh tap water ad libitum. After 3 weeks on a control AIN-93M diet, LNCaP xenografts were initiated in the mice by subcutaneous injections of 2 × 10^6^ cells in 50 μL of phosphate-buffered saline (PBS) plus 50 µL Matrigel (BD Biosciences, Mansfield, MA) in the flank. Mice remained on AIN-93M diet for 7 weeks after cell injection until tumors reached 2–3 cm^3^ in volume and were then sacrificed. Portions of tumor tissues were quickly frozen in liquid nitrogen and stored at −80 °C for mRNA and protein analysis as described below.

### 2.4. RNA Extraction and Sequencing Using RNA-Seq Technology

For gene expression assays, six replicate samples were used for LNCaP cell culture analysis, and six tumor samples were used for each of the two xenograft studies. Total RNA of tumor and cell samples were extracted using TRIzol and then purified using DNase digestion and Qiagen RNeasy columns following the manufacturer’s instructions (Qiagen, Valencia, CA, USA). The integrity of RNA samples was determined using an Agilent Bioanalyzer 2100 (Agilent, Palo Alto, CA, USA). An Illumina RNA-seq sample preparation kit was used to verify the high-quality RNA (Illumina, San Diego, CA, USA). RNA-seq libraries were obtained using an Illumina GAIIx sequencer at 40 bp/sequence with a depth of approximately 24.8 million sequences for each sample.

### 2.5. Data Analysis and Bioinformatics

CLC Genomics Workbench (CLC Bio, Aarhus, Denmark) was used to generate expression values. Reads were trimmed by quality control filters and mapped to the human genome to remove murine contaminants. Total gene expression was quantified using the RNA-seq Analysis tool, allowing no more than two mismatches per reading. Additionally, the mouse reads for housekeeping genes TBP, ACTIN, and GAPDH were 7%, 10%, and 0.3%, respectively, of the total reads. The percentage of mouse cell in our tumor samples may have averaged ~6%; therefore, >90% of the cells in the tumor samples were of human (LNCaP) origin. Mouse sequence was removed before performing the analysis. The data were further analyzed using the methods described below.

#### 2.5.1. Principal Component Analysis

The principal component analysis (PCA) was conducted using default programmed covariance estimation in CLC to identify and visualize differences between treatment groups. The plot was shown in eigenvector with one eigenvalue. Principal components in the plot were grouped by color.

#### 2.5.2. Volcano Plot Analysis

A volcano plot was used to examine the differences/fold changes of the genes from the mean values. Those with significant changes in transcriptome profiles were analyzed using the CLC Genomics Workbench (CLC Bio, Aarhus, Denmark). Differences in genes were shown in a 2D scatter plot. Gene expressions were based on log2 ratio in the x-axis, and the *p*-value was represented as −log10. The genes colored with blue were significantly downregulated (*p* ≤ 0.05; z-score ≤ −2) and the genes colored with red were significantly upregulated (*p* ≤ 0.05; z-score ≥ 2).

#### 2.5.3. Canonical Pathway Analysis of Data Sets

Common differentially regulated genes (with >2X and <−2X cut off) from two xenograft studies were analyzed using the Ingenuity Pathways Analysis (IPA, Ingenuity Systems, and www.ingenuity.com, (accessed on 22 July 2012). The regulation and activation status of pathways were predicted by IPA using z-score and an overlapping *p*-value based on the number of genes, fold-changes, and literature database. Genes or networks with *p*-value less than 0.05 and z-score greater than 2 or less than −2 were considered as significantly altered. Genes were mapped to the Ingenuity Pathways Knowledge Base and overlaid to a global molecular network. The most altered pathways for targets with significance from the IPA library of canonical pathways were determined using the right-tailed Fisher’s test. The *p*-value was used to indicate the probability of correlation between input genes and the IPA canonical pathway reference database.

#### 2.5.4. Network/Pathways Graphical Representation

The networks of pathways were generated using IPA. Upstream and downstream regulators and alterations of genes were graphically represented. Nodes were used to represent genes in networks, and the intensity of nodes indicate the degree of regulation. Genes that were upregulated were marked in red, and downregulated genes were marked in green.

### 2.6. In Vitro Modeling of Androgen Exposure, Cell–Matrix Interaction, Inflammation, and Hypoxia

In vitro experimental models below were used to further elucidate molecular mechanisms that lead to gene/pathway changes.

#### 2.6.1. Effects of Androgen on Gene Expressions

The effects of androgen on LNCaP gene expressions were analyzed as previously described [28]. Briefly, 2.5 × 10^5^ cells/mL of LNCaP were plated in 6-well plates and cultured in RPMI-1640 media with phenol red and 10% FBS. After 24 h, cell media were replaced by RPMI-1640 media containing 10% androgen-depleted charcoal-dextran-stripped serum (CDS) without phenol red. After 24 h, cells were treated with or without 1 nM dihydrotestosterone (DHT) for 48 h. After 48 h treatment, RNA was isolated and cDNA was synthesized as previously described [28] and expressions of target genes were examined using RT-PCR as described below.

#### 2.6.2. Effects of Hypoxia on Gene Expressions

The effect of hypoxia on gene expressions in LNCaP was investigated. Briefly, LNCaP cells were seeded at 2.5 × 10^5^ cells/mL in 6-well plates in RPMI-1640 media with phenol red and 10% FBS. After 24 h, the media was replaced with fresh media containing 150 μM of CoCl_2_. After 48 h treatment, RNA was isolated and cDNA was synthesized as previously described [28], and expressions of target genes were examined using RT-PCR as described below.

#### 2.6.3. Effects of Tumor Cell–Immune Cell Interaction on Gene Expressions

The effects of tumor cell–immune cell interaction on gene expressions in LNCaP cells were tested using conditioned media from the human THP-1 macrophage. Undifferentiated THP-1 (u-THP-1) cells (5 × 10^5^ cells/mL) were plated with differentiation agent PMA (25 ng/mL). After 48 h, u-THP-1 cells were differentiated into d-THP-1 and the culture media were replaced with fresh media +/− 10 ng/mL of LPS for an additional 24 h. A control well with media and without cells was also employed as control. After the incubation period, incubated media were collected and added to LNCaP cells (2.5 × 10^5^ cells/well overnight culture). After 24 h, RNA was isolated and cDNA was synthesized, as previously described [28], and expressions of target genes were examined using RT-PCR as described below.

#### 2.6.4. Effects of Subcellular Matrix on Gene Expressions

For 3D cell culturing, prechilled Matrigel (4 °C overnight) was mixed with LNCaP cells at a concentration of 0.85 × 10^6^ cells/mL (identical to that injected to nude mice to establish the xenograft). The mixture was incubated at 37 °C to allow the Matrigel to gel. After 30 min, 1.5 mL of cell culture media was added to the well. After 72 h, culture media were removed and cells were washed three times with cold PBS. Two milliliters of Corning cell recovery solution were added to cells, and cells were separated from Matrigel. RNA was isolated and cDNA was synthesized as previously described [28], and expressions of target genes were examined using RT-PCR as described below. Gene expression was compared to cells cultured in-well without Matrigel.

### 2.7. Real-Time PCR Analysis of Gene Expression

Real-time PCR was used to quantify differences in relative mRNA levelsBriefly, 1 µg of total RNA was used for cDNA synthesis using the AffinityScript Multi-Temperature cDNA Synthesis kit according to manufacturer’s protocol. Real-time PCR was performed using the TaqMan Fast Universal PCR Master Mix following the previously published protocol [28]. TaqMan gene expression assay was used to detect mRNA levels of AHR (Hs00169233_m1), AR (Hs00171172_m1), CDH1 (Hs01023895_m1), CXCL12 (Hs00171022_m1), CXCR7 (Hs00604567_m1), F3 (Hs01076029_m1), FLNA (Hs0092465_m1), ITGA6 (Hs01041011_m1), PLA2G2A (Hs00179898_m1), PHLDA1 (Hs00378285_g1), UGT2B15 (Hs00870076_s1), UGT2B28 (Hs00852540_s1), ZEB2 (Hs00207691_m1), RhoB (Hs03676562_s1), and ITGA1 (Hs00235006_m1). TATA-binding protein (Tbp) (Hs00427620_m1) was used as the housekeeping gene for normalization. Relative expression value was generated using the ΔΔCt method as described previously [28].

### 2.8. Statistical Analysis

The false discovery rate (FDR) adjusted *p*-value (Student’s *t*-test) was used to identify the significance in bioinformatic data analysis, and a value less than 0.05 was considered significant (CLC Bio, Aarhus, Denmark). For pathways analysis using IPA, z-score was used to predict the activation or inhibition of biological functions. Pathways with a z-score greater than 2 were considered as significantly activated, and that less than −2 were considered as significantly inhibited (IPA, Ingenuity Systems, and www.ingenuity.com). For in vitro experiments, Prism 8.3 (GraphPad Prism, San Diego, CA, USA) was used. For multiple group comparison, ANOVA followed by post hoc test was used. For two-group comparison, an unpaired *t*-test was used. *p* < 0.05 is considered significantly different.

## 3. Results

### 3.1. Global Transcriptomic Comparison of Parent LNCaP Cells and LNCaP Cell Tumor Xenograft

#### 3.1.1. Principal Component Analysis of Cultured LNCaP Cells vs. LNCaP Xenograft Tumor

**Figure 1:**
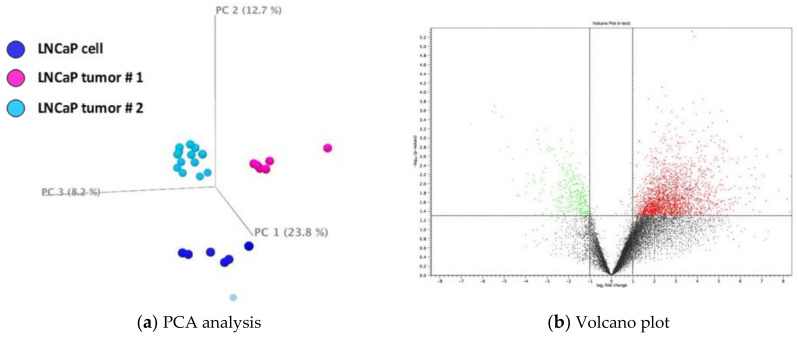
(**a**) Principal component analysis (PCA) and volcano plot of gene expression data comparison. A. PCA of xenograft tumor (n = 6 for each experiment) as compared to LNCaP cells (n = 6). Sets of tumor samples are colored in cyan (#1) and light blue (#2) and cultured cell samples are colored in dark blue. (**b**) Volcano plot of transcript profiles in xenograft tumor as compared to LNCaP cells. Transcriptome profiles with z-score less than −2 are marked in green and with z-score greater than 2 marked in red.

To obtain a probabilistic interpretation of LNCaP xenograft (n = 6 for each experiment) and cell samples (n = 6), principal component analysis (PCA) was performed. Transcriptomic profile of the cultured parent LNCaP cells was analyzed and compared to the LNCaP xenograft tumor samples (Figure 1a). The PCA result indicated samples within each experimental group clustered together. Tumor samples (cyan) were generally well-separated from the cell samples (dark blue) in the vertical direction. We also compared cell samples to a previous dataset of tumor sample (tumor sample #2, light blue) available in the laboratory. They are also well-separated from each other. However, the two tumor sample sets appeared to be distinct from each other. Hence, for IPA analysis described below, a list of genes (1568 genes, Supplementary Materials 1) commonly changed in both tumor sample sets was used for analysis.

#### 3.1.2. Volcano Plot of Cultured LNCaP Cells vs. LNCaP Xenograft Tumor

The changes of global transcriptomic profiles in LNCaP xenograft tumor as compared to LNCaP cultured cells were analyzed using a volcano scatter plot. Figure 1b illustrates the comparison between tumors from experiment #1 and parent LNCaP cells. There appear to be more upregulated genes (red dots) in LNCaP xenograft tumor than downregulated genes (green dot).

#### 3.1.3. Comparison of Gene Expression in Cultured LNCaP Cells vs. LNCaP Xenograft Tumor Using IPA

As noted above, 1568 genes commonly identified in tumor sample 1 and 2 were imported for IPA analysis (Supplementary Materials 1). After using >2x and <−2x criteria, there are 1124 analysis-ready molecules, 637 downregulated and 487 upregulated, generated by IPA analysis. Based on their fold change, Table 1A,B lists the top 10 genes with higher and lower expressions in tumor samples compared to cultured parent LNCaP cells. The four genes with higher expression levels in LNCaP xenograft tumors, F3, CREB3L1, ORM1, and RGS2, were increased by 210.04, 182.71, 174.68, and 138.16-fold, respectively, as compared to LNCaP cultured cells. Among the genes with higher expression, F3 gene encodes coagulation factor III, which is a cell surface glycoprotein that enables cells to initiate the blood coagulation cascades [29]. CREB3L1 are involved in cell migration and proliferation [30]. ORM1 plays a role in infection and inflammation [31]. The expression of RGS2 is associated with the lower expression of androgen-independent AR [32]. The genes TIMP1, MAGEB17, CA3, and KRT75 expressions are relatively lower with sequence read at <100 in both cultured cells and tumor samples. TIMP1, TIMP Metallopeptidase Inhibitor 1, belongs to the TIMP (tissue inhibitors of metalloproteinase) gene family [33,34]. The proteins encoded by this gene family are natural inhibitors of the matrix metalloproteinases (MMPs), a group of peptidases involved in degradation of the extracellular matrix [33,34]. In addition to its inhibitory role against most of the known MMPs, the encoded protein is able to promote cell proliferation in a wide range of cell types, and may also have an antiapoptotic function [33,34]. Transcription of this gene is highly inducible in response to many cytokines and hormones [33,34]. MAGEB17, MAGE Family Member B17 function is relatively unclear. CA3, Carbonic anhydrase III (CAIII) is a member of a multigene family (at least six separate genes are known) that encodes carbonic anhydrase isozymes [35]. These carbonic anhydrases are a class of metalloenzymes that catalyze the reversible hydration of carbon dioxide and are differentially expressed in a number of cell types [35]. The expression of the CA3 gene is strictly tissue specific and present at high levels in skeletal muscle and much lower levels in cardiac and smooth muscle [35]. KRT75, Keratin 75, is a member of the type II keratin family, clustered on the long arm of chromosome 12. Type I and type II keratins heteropolymerize to form intermediate-sized filaments in the cytoplasm of epithelial cells [36]. This gene is expressed in the companion layer, upper germinative matrix region of the hair follicle, and medulla of the hair shaft [36]. The encoded protein plays an essential role in hair and nail formation [36]. UNC80, which is also known as C2orf21, encoded protein belongs to a component of a voltage-independent ”leak” ion-channel complex [37]. This gene performs essential functions, such as serving as a bridge between two other components (sodium leak channel nonselective and UNC79) and as a scaffold for Src kinases [37]. Leak channels are known to play an important role in the establishment and maintenance of resting membrane potentials in neurons [37].

As for the top 10 genes with lower expressions in tumors rather than cultured cells, FCAMR’s expression level was one of the lowest in tumors, by −62.57-fold compared to cultured cells (Table 1B). FCAMR has been reported to be involved in the regulation of immune responses [38]. In addition, 4 out of 10 top genes with lower expressions in LNCaP tumors, including UDP-glucuronosyltransferase 2B15 (UGT2B15), UGT2B17, UGT2B10, and UGT2B28, are from the UDP-glucuronosyltransferases family [39]. The reaction catalyzed, known as glucuronidation, is an intermediate step in the metabolism of steroids and xenobiotics [39]. These genes play pivotal roles in the regulation of androgen metabolism [40]. The UGT2B15, UGT2B17, UGT2B10, and UGT2B28 enzymes exhibited lower expressions, by −62.32, −54.14, −35.44, and −20.85-fold, respectively, as compared to cultured cells. UGT2B15 and 17 are the major genes based on sequence reads. UGT2B15 encodes a glycosyltransferase that is involved in the metabolism and elimination of toxic compounds, both endogenous and of xenobiotic origin [39,40,41]. This gene was reported to play a role in the regulation of estrogens and androgens [30,31,32,33,34,35,36,37,38,39,40,41]. UGT2B17 encoded an enzyme that catalyzes the transfer of glucuronic acid from uridine diphosphoglucuronic acid to a diverse array of substrates including steroid hormones and lipid-soluble drugs [39,40,41]. UGT2B 10, 11, and 28 also act on a diverse array of substrates including steroid hormones and lipid-soluble drugs [39]. TP53INP1, Tumor Protein P53 Inducible Nuclear Protein 1, is an antiproliferative and proapoptotic protein involved in cell stress response that acts as a dual regulator of transcription and autophagy [42]. ID3, Inhibitor of DNA Binding 3, is a helix–loop–helix (HLH) protein that can form heterodimers with other HLH proteins [43]. ID3 was reported to be implicated in regulating a variety of cellular processes, including cellular growth, senescence, differentiation, apoptosis, angiogenesis, and neoplastic transformation [43]. HRNR (Hornerin) and S100A7 (S100 Calcium Binding Protein A7) were low in expression with <100 reads. HRNR is a component of the epidermal cornified cell envelopes [44]. Both proteins encode a gene that is a member of the S100 family of proteins containing 2 EF-hand calcium-binding motifs. S100 proteins are localized in the cytoplasm and/or nucleus of a wide range of cells, and are involved in the regulation of a number of cellular processes such as cell cycle progression and differentiation [45]. S100 genes include at least 13 members that are located as a cluster on chromosome 1q21. This protein differs from the other S100 proteins of known structure in its lack of calcium-binding ability in one EF-hand at the N-terminus [45]. The protein is overexpressed in hyperproliferative skin diseases, exhibits antimicrobial activities against bacteria and induces immunomodulatory activities [45].

Together, the top genes with higher and lower expressions in LNCaP xenograft tumor compared to LNCaP cultured cells indicate alterations of genes involved in the regulation of androgen, immune response, cell migration, proliferation, and metabolism in the LNCaP tumor.

### 3.2. Elucidating Pathway Differences between LNCaP Xenograft Tumor and Cultured LNCaP Cells

IPA analysis was used to identify pathways and associated gene networks that were altered in tumor samples compared to the cultured cells. Additionally, we also query specific pathways that are related to prostate carcinogenesis, and the results are described below.

#### 3.2.1. Top Canonical Pathways Differences Identified from IPA Analysis

The top canonical pathways identified by IPA based on *p*-value (Supplementary Materials 2) are listed in Table 2A. The aryl hydrocarbon receptor-related pathway is the top canonical pathway identified, with 15% of the genes (21/137) in the pathway altered. Additionally, the pathway was calculated with a negative z-score, indicating an overall inhibition of this pathway. Genes associated with the pathways are in Supplementary Materials 2, and, as expected, several of the xenobiotic metabolism-related genes such as ALDHs and NQO1 were downregulated. AHR is associated with a wide range of cellular function, including xenobiotic metabolisms, cell-cycle progression, apoptosis, and more that are related to tumorigenesis (Supplementary Figure S1a). In contrast, the HOTAIR regulatory pathway, which was also identified as one of the top canonical pathways, has a positive z-score; suggesting an overall upregulation of the pathway. HOTAIR, a lncRNA, has been reported to enhance the androgen receptor-mediated transcriptional program and drive castration-resistant prostate cancer [46]. Prominent genes associated with the pathway include AR and cell-matrix interaction genes such as CDH1 and ICAM (Supplementary Materials 2). Metastasis and tumor progression are among the functions related to the pathway (Supplementary Figure S1b). Using a threshold of *p* < 0.05 as cutoff, Table 2 lists the top–down and upregulated canonical pathways based on the z-score. Table 2 was presented using a *p*-value ranking, as we did not find upregulated pathways with a z-score >2. Overall, there appeared to be significant changes in several signal transduction pathways in the tumor as compared to cultured cells. The predicted molecular and cellular function associated with altered pathways are listed in Table 3. Genes associated with the pathways are listed in Supplementary Materials 3. Not surprisingly, cellular growth, proliferation, death, and survival are the top functions altered. However, cell movement and assembly also emerged as top altered functions.

#### 3.2.2. Identification of Top Networks Differences

Using IPA analyses, we organized our data set into 25 interconnected networks (Supplementary Figure S1a). The network list, genes associated with each network, and network functions are in Supplementary Materials 3. Some networks interact with more networks (Supplementary Figure S2) than others. In addition, some genes are associated with multiple networks, indicating they may be serving multiple roles/functions within the cell (Supplementary Materials 3). AR is associated with network #3 (Supplementary Figure S2 #3). Genes in network #3 include some of the top downregulated genes belonging to the UGT2B family. AHR, which is associated with the top canonical pathway identified, is associated with network #2 and #18 (Supplementary Figure S2 #2 and 18). Notably, there appeared to be less interaction in network #18 between AHR and other components within the network than #2. Thus, AHR either directly or indirectly interacts with several epithelial mesenchymal transition (EMT)-associated genes such as CDH1, SNAI1, and TWIST1 [47].

#### 3.2.3. Identification of Top Upstream Regulators and Top Causal Networks

**Figure 2:**
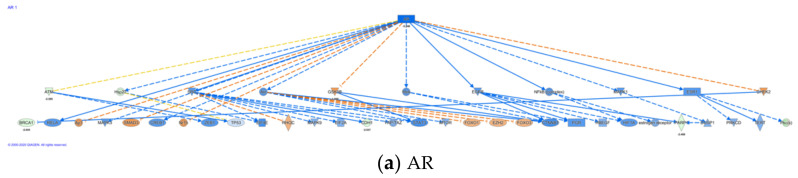

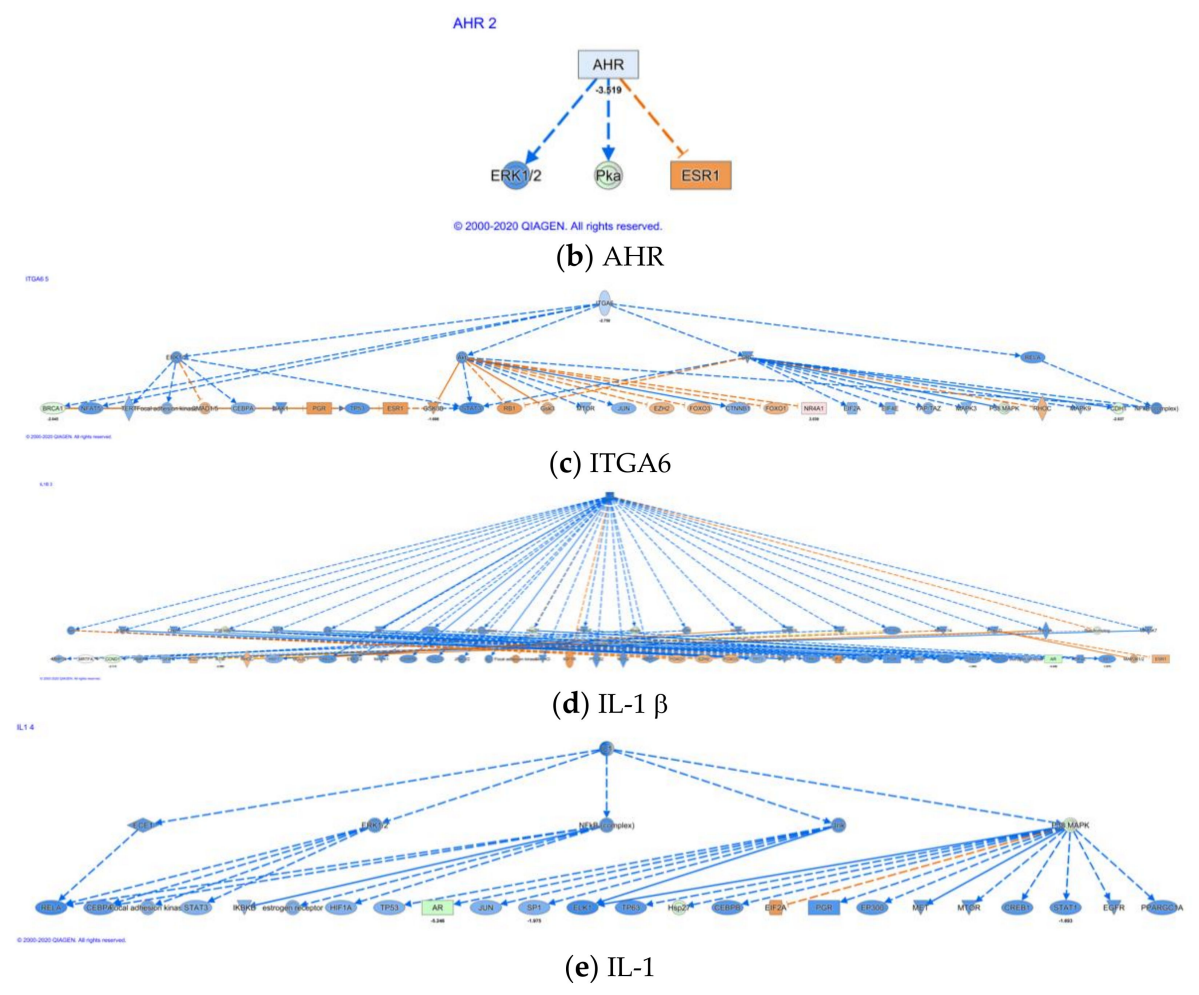
Casual network for AR, AHR, ITGA6, IL-1β, and IL-1. Casual network analysis was performed using IPA as described in Materials and Methods. The diagram depicts interaction with other regulators: (**a**) AR, (**b**) AHR, (**c**) ITGA6, (**d**) IL-1β, and (**e**) IL-1.

IPA also identifies upstream regulators (Supplementary Materials 4) that are involved in conferring differential expression. A total of 338 upstream regulators (Supplementary Materials 4) were identified to be different between parent LNCaP cell and LNCaP xenograft tumors. Among the upstream regulators, 40 regulators were identified with a mechanistic network associated with these upstream regulators (Supplementary Materials 4). Five cytokines/growth factors (TNF, TGFb1, NRG1, EGF, IGF) and nine kinases, including EGFR were identified as upstream regulators. In addition, 14 transcriptional factors and among them three hormone receptors: PGR, AR, ESR1, were also identified as upstream regulators. There were also 360 causal networks identified to be different between parent LNCaP cell and LNCaP xenograft tumors (Supplementary Materials 5). Among the causal network, 32 network master regulators have expression data from our current data set. Some of the master regulators, such as AR, are associated with multiple networks (Supplementary Materials 5). Top upregulated master regulators in the tumor, as compared to the parent cells, include ZEB2 and PHLDA1; while top downregulated master regulators include AR, AHR, and ITGA6. The causal networks associated with AHR, AR, and ITGA6 and their downstream regulators are illustrated in Figure 2a–c, respectively. Further analysis indicates that there were 82 causal networks predicted to be inhibited while only 14 were predicted to be activated in tumor vs. parent cells. One feature apparent from the causal networks analysis was that there are shared genes between the networks. For example, ABCG1, a cholesterol efflux transporter associated with maintenance of cholesterol homeostasis, appeared in several networks [48]. This is indicative of multiple signals that may feed into the regulation of this gene.

Network analysis described above (Supplementary Materials 3) and analysis of causal networks (Supplementary Materials 5) using IPA highlighted the effects of altering the AR-dependent pathway. Androgen receptor (AR) exhibited lower expression (~−5×) in the LNCaP xenograft tumor compared to cultured cells (*p* < 0.05). As shown in the network of AR regulation (Supplementary Figure S2 #3), downregulation of AR can lead to expression changes of multiple proteins, such as UGT2B28, a highly downregulated gene (~−20×) described above. Interestingly, most downregulated or upregulated genes in the pathway tend to be modest at ~2–3×. Of note, PMEPA1, a gene upregulated 3.7-fold, was identified as regulated by AR. Expression of this gene is induced by androgens and transforming growth factor beta [49], and the encoded protein suppresses the androgen receptor and transforming growth factor beta signaling pathways through interactions with Smad proteins [49]. Overexpression of this gene may play a role in multiple types of cancer [49]. This gene is also involved in downregulation of the androgen receptor (AR), enhancing ubiquitination and proteasome-mediated degradation of AR [49]. Consistently, TGFb1 was also identified as top upstream regulator and causal network with an effect on AR (Supplementary Materials 4 and 5).

As mentioned above, AHR is the top canonical pathway identified to be altered. Networks (Supplementary Materials 3, Supplementary Figure S1a) and causal network analysis (Supplementary Materials 5) indicate downregulation of AHR (~3×). A prominent feature of this pathway is downregulation of SNAI1 and CDH1, which are involved in EMT (Supplementary Figure S2, #2), suggesting a role for a AHR-mediated pathway in such regulation.

#### 3.2.4. Alteration in Cytokine- and Chemokine-Related Networks

In the causal network analysis, there were several cytokines identified as master regulator (Supplementary Materials 5). One of the top master regulators identified was IL-1b/IL-1. A total of 59 target regulators was identified downstream (Figure 2d,e). At least 180/142 target molecules were associated with the IL-1b/IL-1 causal network (Supplementary Materials 5) and served a wide range of cellular functions, including cell proliferation, DNA damage/repair, and androgen-mediated pathways. Notable target genes associated with these networks include AR and AHR (Supplementary Materials 5). Additionally, the chemokine CXCL12 and its decoy receptor CXCR7 (identified as ACKR3 in IPA) were also among the purported target molecule. Our previous work indicates a role of chemokine CXCL12 and its receptors CXCR4/7 in migration of LNCaP cells [28]. CXCR7/ACKR3 appeared to serve as a decoy receptor to counter CXCR4-mediated effect elicited by CXCL12. CXCL12 and its receptor CXCR7 (Table 4, *p* < 0.05) were the only chemokine and receptor observed with significant alterations in the xenograft model as compared to the parent LNCaP cells. Moreover, CXCR4 was identified as master regulator in our causal network analysis (Supplementary Materials 4,5). Prominent molecules identified in this network included aforementioned ABCG1, AHR, and CDH1, suggesting interplay between the various networks. Additionally, CXCR7/ACKR3 is associated in several causal networks and its target molecule include IL-1 and AR as master regulators.

#### 3.2.5. Genes/Pathways Alteration Associated with Cell–Cell Matrix/Adhesion

Cancer progression is in part determined by cell–cell matrix interactions [50], which is a major difference between parent cell in culture vs. tumor xenograft. Moreover, during the progression of prostate cancer, integrins were reported to modulate various cell functions, including cell migration, survival, and aberrant cellular growth [51]. Therefore, we also query specific genes and pathways related to cell matrix/adhesion. Integrin and adhesion related genes including ITGA2, ITGA6, and ICAM5 were changed by −6.12, −2.75, and −5.77-fold, respectively, comparing tumor to parent cells (Table 5). In contrast, ICAM1 was 5.48-fold higher in tumor samples (Table 5). FN1 was lower in tumor samples by −9.6. Comparing tumor to parent cell, the cadherins CDH1 and CDH11 were different by −2 and 11.3-fold. The MMPs, peptidase that shapes the extracellular matrix [52], were also different. The MMPs changed include MMP 7, 10, 16, and 19. The fold differences were 13, 14.5, −4.97, and 7.0, respectively.

Additionally, network analysis identified ITGA6, ITGA2, which are associated with network #11 (Supplementary Figure S2, #11 network). Some notable features of the network include PHLDA1 as a member. PHLDA1 was described above as a top upregulated, upstream master regulator (Supplementary Materials 4) of causal network. ITGA6 was also identified as master regulator of a causal network (Figure 2c). Molecular targets in this causal network included CXCR7/ACKR3, AHR, AR, CDH1, FN1, ICAM1, and MMPs. In addition, ZEB2, another highly altered upstream regulator of a causal network mentioned above, is associated with network #2, consisting of CDH1 as a member molecule, as well as AHR (Supplementary Figure S2, network #2). Furthermore, MMPs appeared to be associated with multiple causal networks and may subject to diverse regulation.

### 3.3. Validation of Gene Expressions in LNCaP Xenograft Tumors Compared to Cultured Cells

**Figure 3:**
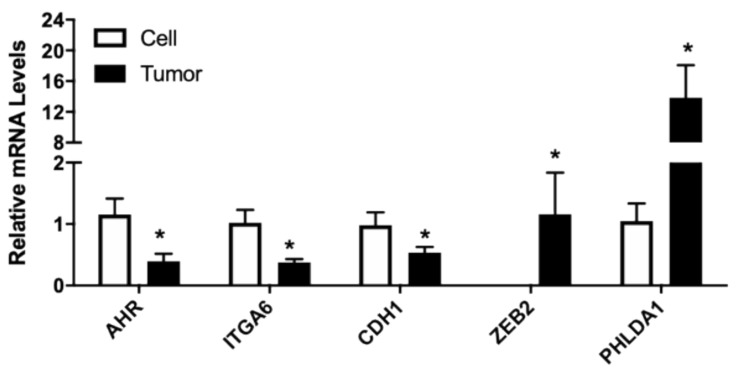
Relative mRNA levels of selected genes in cultured LNCaP cells and LNCaP xenograft tumors. RNA was isolated from culture LNCaP cell and LNCaP tumor xenograft and gene expression determined using real-time RT-PCR, as described in Materials and Methods. Results were normalized to the cultured cell control and expressed as relative mRNA levels (mean ± SD, n = 15 for cells and 18 for tumor samples); *p*-values ≤ 0.05 were considered as significant and * indicates significantly different from control.

The changes of selected genes in LNCaP xenograft tumor compared to the cultured cells using RNA-seq were further validated using RT-PCR. AR, AHR, CDH1, CXCL12, CXCR7, F3, FLNA, ITGA6, PHLDA1, UGT2B15, and ZEB2 were analyzed in cultured cell and tumor samples. Compared to the parent LNCaP cells, expression of AR, AHR, CDH1, CXCR7, FLNA, ITGA6, and UGT2B15 in tumor was significantly inhibited (Figure 3). The mRNA level of CXCL12, F3, PHLDA1, and ZEB2 was upregulated in the tumor samples (Figure 3). Overall, changes in selected genes were consistent with the RNA-seq findings, further confirming transcriptome changes from the RNA-seq analysis.

### 3.4. The Effects of In Vitro Environments on LNCaP Gene Expressions

As described above, many potential signals may be involved in conferring differential gene expression between the parent LNCaP cells and xenograft tumor. To further delineate mechanisms that may result in differential gene expression in the parent LNCaP cells and tumor, we also modeled regulation of gene expression under in vitro conditions that included exposure to androgen, hypoxic condition, interaction with matrix gel, and interaction with immune cells. The following sections describe the results.

#### 3.4.1. Genes Responsive to Androgen

**Figure 4:**
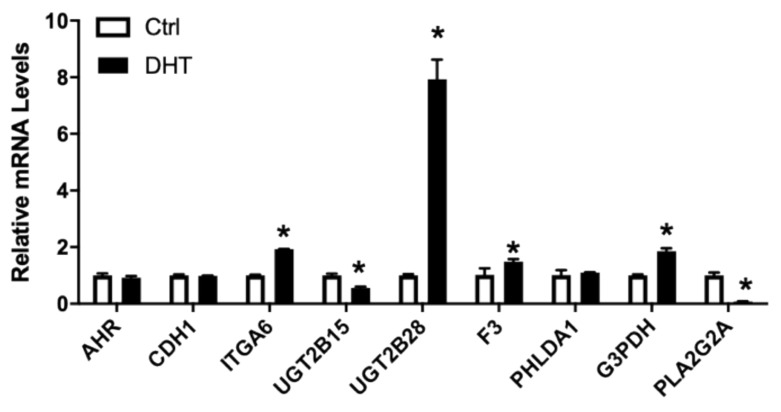
Relative mRNA levels of selected genes in LNCaP cells treated without/with 1 nM DHT. LNCaP cells were treated with 1nM DHT, RNA isolated, and the gene expression analyzed, as described in Materials and Methods. Results were normalized to the untreated control and expressed as relative mRNA levels (mean ± SD, n = 3); *p*-values ≤ 0.05 were considered as significant and * indicates significantly different from control.

Androgens play a critical role in prostate physiology and prostate cancer carcinogenesis [53]. Exposure of LNCaP cells to 1 nM DHT in culture lead to lower expressions of CXCR7, AR, UGT2B15 (Figure 4). In contrast, ITGA6 was upregulated ~2x and a slight but significant upregulation of F3 and FLNA (Figure 4) was also observed. AHR, CXCL12, CDH1, PHLDA1, and ZEB2 were not affected by DHT treatments. We also confirmed that PLA2G2A, identified as an upregulated gene in the tumor and directly regulated by AR in network #3, to be inhibited by DHT treatments in the cultured LNCaP cells (Figure 4).

#### 3.4.2. Genes Associated with Hypoxia

**Figure 5:**
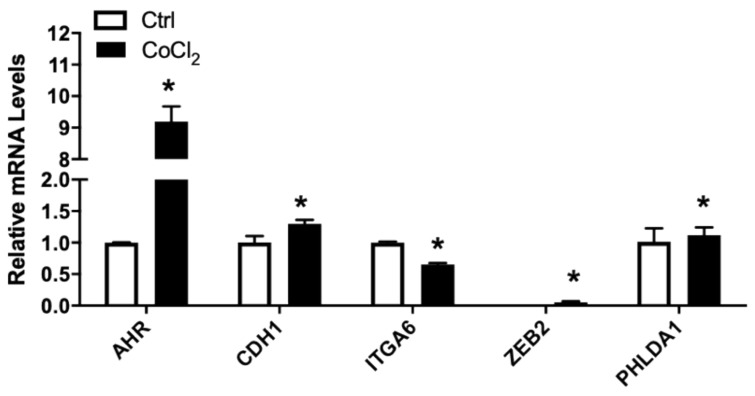
Relative mRNA levels of selected genes in LNCaP cells treated without/with CoCl_2_. LNCaP cells were subjected to CoCl_2_-induced hypoxia, RNA isolated, and the gene expression analyzed, as described in Materials and Methods. Results were normalized to the untreated control and expressed as relative mRNA levels (mean ± SD, n = 3); *p*-values ≤ 0.05 were considered as significant and * indicates significantly different from control.

Hypoxia is one of the hallmark conditions in solid tumor [54]. Hypoxic condition was induced in vitro by treating cells with 150 µM CoCl_2_ [55]. The relative mRNA levels of AR, ITGA6, UGT2B15, and to a lesser extent, CXCR7, were all significantly reduced by exposure to CoCl_2_ (Figure 5). In contrast, AHR, FLNA, PHLDA, and to a lesser extent, CDH1, were upregulated by CoCl_2_ treatments (Figure 5). The treatment of LNCaP cells with CoCl_2_ had no influence on the mRNA levels of CXCL12 or F3 in LNCaP cells.

#### 3.4.3. Genes Associated with Subcellular Matrix

**Figure 6:**
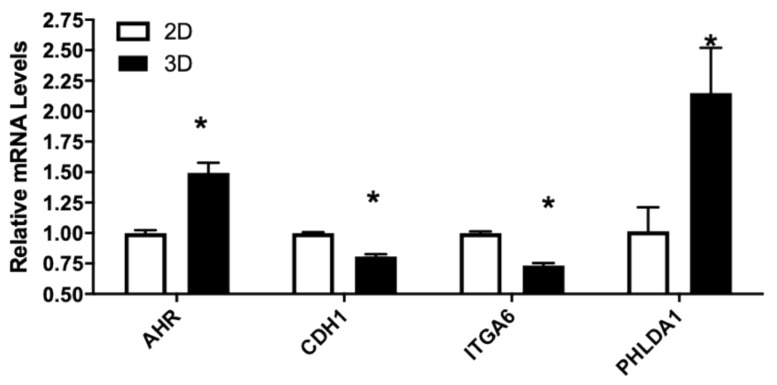
Relative mRNA levels of selected genes in LNCaP cells culture in 2D or 3D conditions. LNCaP cells were cultured in 2D or 3D conditions as described in Materials and Methods. RNA were isolated and gene expression determined using real-time RT-PCR, as described in Materials and Methods. Results were normalized to the untreated control and expressed as relative mRNA levels (mean ± SD, n = 3); *p*-values ≤ 0.05 were considered as significant and * indicates significantly different from control.

The LNCaP tumor xenograft was created using Matrigel as subcellular matrix. To elucidate potential effects of cell–cell matrix interaction on gene expression, LNCaP cells were grown either in 2D tissue culture plastic wells or seeded in 3D Matrigel matrix. As shown in Figure 6, CXCL12 mRNA levels and to a lesser extent CXCR7, AR, CDH1, ITGA6, and UGT2B15 were all significantly downregulated in LNCaP cultured in Matrigel. PHLDA1 and to a lesser extent AHR were upregulated in 3D culture compared to 2D culture. In addition, no change in F3 or FLNA mRNA levels was observed in LNCaP growing in either 2D or 3D culture conditions.

#### 3.4.4. Genes Associated with Tumor Cell–Immune Cell Interaction

**Figure 7:**
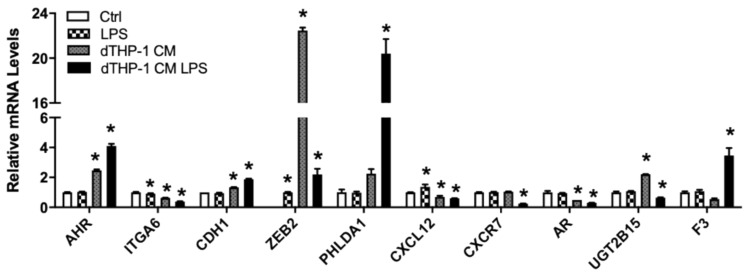
Relative mRNA levels of selected genes in LNCaP cells treated with THP-1 macrophage conditioned media. LNCaP cells were cultured in RPMI-1640 media with or without LPS, and THP-1 macrophage-conditioned media with or without LPS, as described in Materials and Methods. RNA isolation and gene expression were determined using real-time RT-PCR, as described in Materials and Methods. Results were normalized to the untreated control and expressed as relative mRNA levels (mean ± SD, n = 3); *p*-values ≤ 0.05 were considered as significant and * indicates significantly different from control.

Tumor cells interact with surrounding stromal cells, including immune cells, which consist of the tumor environment that can influence tumor development [56]. The Nu-Nu mice, although lacking T cells, are known to have enhanced macrophage response [57]. We used LNCaP cells grown in conditioned media from the PMA-differentiated human THP-1 macrophage, stimulated with or without the bacteria cell wall polysaccharide LPS, to mimic potential interaction between macrophage and LNCaP cells. Interestingly, the conditioned media enhanced expression of AHR, CDH1, ZEB2, and UGT2B15 (Figure 7). AHR and CDH1 expression were further enhanced in the media from macrophage stimulated with LPS but not ZEB2 and UGT2B15. PHLDA1 and F3 were induced only in media from LPS-stimulated macrophage (Figure 7). In contrast, ITGA6, CXCL12, and AR were downregulated by conditioned media and the inhibitory effects were further enhanced in LPS-stimulated macrophage conditioned media (Figure 7). CXCR7, on the other hand, was downregulated only in LPS-stimulated macrophage conditioned media (Figure 7).

## 4. Discussion

The current study seeks to use the NGS approach coupled with complementary in vivo and in vitro models to (1) provide molecular information on the progression of human prostate cancer cells in vivo, (2) identify possible tumor microenvironmental signals that lead to these molecular changes, and (3) facilitate molecular target identification to elucidate the mechanism of action for dietary prostate cancer prevention studies. It was not surprising that some genes will change in the more complex interactions existed in vivo, which are not captured in cell culture environment. However, we were surprised by the number of different genes between the tumors and the parent cells in culture. We identified greater than 1000 genes that changed 2× or greater in the tumor xenograft vs. culture cells. These data suggest that the tumor microenvironment may play a very substantive role in tumorigenesis. More importantly, due to the interactive nature of cellular networks identified in our analysis, some cellular interactions may not be replicated in culture cell models. Another surprise for us was observing a ~20% difference between two separate xenograft studies. This may contribute to interlaboratory variability in observing gene expression changes and influence one’s transcriptomic analytic interpretation and extrapolation to biological effects. Hence, understanding the limitation of different cancer models and approaches to better define experimental questions is critical in providing physiologically relevant data.

One of our foci was to first query well-known pathways related to prostate carcinogenesis and ask how these pathways change. While the top downregulated pathways were identified with *p* < 0.05 and z-score >2, the impact on upregulated pathways appeared less dramatic. These results suggest that the changes in the top upregulated pathways may be less important overall during the development of xenograft tumors. AR and AHR were among the several prominent pathways that changed in the LNCaP tumors vs. the LNCaP culture cells. AR mediates the effects of androgen, and exposure to androgen is a known risk factor for prostate carcinogenesis [53]. Progressing to an androgen-independent state is one of the hallmarks during prostate carcinogenesis [53]. Our analyses indicate a downregulation of AR expression and an overall inhibition of AR-mediated pathways in the tumor samples vs. culture cells. This supports LNCaP cells progressing to an androgen-independent state in the xenograft. Furthermore, our in vitro study indicated three factors particularly relevant in contributing to the downregulation of AR-mediated pathways: exposure to androgen, hypoxia state of the tumor environment, and interaction with immune cells. Although cytokines such as IL-6 and IL-8 (CXCL8) have been proposed to be involved in the process [58], it would seem other factors may also play a role. Our bioinformatic analysis using IPA also identifies IL-1 as a candidate signal that may contribute to androgen-independence. Causal pathway analysis suggests the effect of IL-1 may be indirect and mediated through regulation of JNK and/or MAP kinases. IL-1 is also known to contribute to angiogenesis [59] and inflammatory environment [60]. Hence, these results highlight the importance of IL-1 in prostate cancer progression, which may derive from regulation of several pathways, to promote tumor growth and progression. Network analysis also suggests the lncRNA HOTAIR-mediated event [61] as a potential pathway that regulated AR. These data provide additional insights to support multiple mechanisms that may contribute to prostate cancer cell’s progression to androgen-independence. The AHR-related pathway is another pathway that was inhibited in the tumor xenograft. The AHR pathway is known to be involved not only in the disposal of carcinogens but more recently in immune responses [62]. The inhibition of AHR could lead to decreased disposal of toxic compounds, including reactive oxygen species generated within cell or from tumor microenvironment. This may result in further DNA damage and tumor progression to more malignant phenotype. AHR appeared to be subjected to regulation by inflammatory environment and hypoxia. Both conditioned media and LPS-stimulated condition media, as well as CoCl_2_ treatment, lead to induction of AHR. These effects appeared to be different from downregulation of AHR observed in tumors. However, it is known that feedback regulation of the AHR pathway exists [63]; therefore we reason that prolonged inflammatory exposure and hypoxic conditions may lead to downregulation of AHR, thus shutting down the AHR pathway.

Two genes, ZEB2 and PHLDA1 (TDAG51), were identified as novel top regulated genes and master regulator of pathways. The pathways appeared to be associated with cell–cell matrix interaction. Although ZEB2 was detectable in tumor samples, it was at below the limit of detection in cultured cells; therefore we were unable to test the effects of various conditions on ZEB2 in the cell study. PHLDA1 (TDAG51), on the other hand, was detectable in cultured cells. PHLDA1 was reported to be induced by cell interaction with extracellular matrix and related to cell apoptosis [64]. Consistently, our results from interaction with cell matrix as model by culturing cells in Matrigel, indicated cell–cell matrix interaction as the main inducer of PHLDA1. In addition, inflammation as modeled by LPS-stimulated macrophage conditioned media also induced PHLDA1, supporting a role for PHLDA1 in inflammatory response. The gene ITGA6 was identified as a target for PHLDA1. Consistently, both interactions with cell matrix and inflammation also lead to downregulation of ITGA6. ITGA6 itself is also a master regulator of a causal pathway that includes target molecules such as AHR, AR, and CXCR7/ACKR3, thus connecting PHLDA1 with androgen, xenobiotic metabolisms, and immune responses. We therefore hypothesized that cell–matrix interaction and inflammation may act through PHLDA1 to influence tumorigenesis. Further elucidation of whether or not PHLDA1 plays a critical role in tumorigenesis is warranted. However, our results on ITGA6 expression appeared to be in contrast with another report indicating the expression of ITGA6 is associated with metastatic phenotype [65]. It is possible that additional regulation or changes during prostate carcinogenesis may contribute to such a difference and warrant further elucidation. Changes in genes are also reflective of metabolic condition within the tumor microenvironment. For example, the highly upregulated gene CA3 is involved in carbon dioxide metabolisms. Given the hypoxic nature of the tumor, upregulation of these enzymes is consistent with requirements to deal with generation of CO_2_.

Analysis of top expressed genes also provide some insights into potential mechanisms involved in prostate carcinogenesis. The coagulation factor III (F3) was the gene with highest expression in LNCaP tumor xenograft as compared to cultured cells. F3 could be activated by immune stimuli or inflammation associated genes such as lipopolysaccharide (LPS), TNF, VEGFA, F2, phorbol-myristate-acetate (PMA), and IL-1β (Genecards, NCBI). The upregulation of F3 in the tumor sample serves as a marker to indicate a proinflammatory environment within the xenograft. Our in vitro data observing an upregulation of F3 in LPS-stimulated macrophage-conditioned media provide further support for this hypothesis. Although we did observe upregulation of F3 expression in DHT, the magnitude of change was small, therefore we consider inflammation may be the primary inducer of F3. The gene with the second highest expression, CREB3L1, has been reported to be regulated by intramembrane proteolysis in response to virus infection [66]. The upregulation of this gene would also support inflammatory microenvironment in the tumor xenograft. The third highest expressed gene, ORM1, encodes acute phase plasma protein. The upregulation of ORM1 is also associated with acute inflammation [67]. The function of this gene has not yet been fully understood, but ORM1 have reported that this gene might be involved in immunosuppression [68]. Similar to F3 and CREB3L1, the upregulation of ORM1 (by 174.684-fold) indicated that the progression of prostate cancer in LNCaP xenograft tumor might be driven by an inflammatory tumor microenvironment. Given the proposed immunosuppressive properties of ORM1, we hypothesize that an increase in ORM1 may allow cancer cells to escape immune surveillance, which warrants further study. In addition, it should be noted that the fourth most upregulated gene, RGS2, is associated with the downregulation of androgen-independent AR activity [32]. The increase of RGS2 expression might contribute to the decreased mRNA level of AR. The end results would be inhibition of the AR-mediated pathway and is consistent with the development of an androgen nonresponsive phenotype of the xenograft. For the top genes with lower expressions in the tumors, Fc alpha/mu receptor (FCAMR) was a novel gene identified with one of the lowest expression values in LNCaP tumor xenograft compared to LNCaP cells (Table 1B). FCAMR is a receptor that has dual specificity for IgA and IgM [69]. FCAMR binds to IgA and IgM and may be responsible for mucosal immunity [70]. In the case of IgA, known to be produced by prostate epithelial cell, FCAMR may be responsible for the secretion of IgA. The downregulation of FCAMR might be associated with the dysregulation of immune system in the prostate cancer. A group of UGT2B enzymes were found to be significantly lower in tumor xenografts. UGT2B15 and UGT2B17 were the second lowest expressed genes. Together with UGT2B10 and UGT2B28, these four genes belong to the UDP-glucuronosyltransferase family [40]. UGT2B15, UGT2B10, UGT2B17, and UGT2B28, were reported to be critical regulators for androgen metabolism [40]. Chouinard et al. [41] reported that both UGT2B15 and UGT2B17 enzymes are major determinants of the androgen response in prostate cancer LNCaP cells. UGT2B15 and UGT2B17 enzymes conjugate dihydrotestosterone (DHT) and metabolites of DHT, which includes androstane-3α, 17β-diol (3α-DIOL), and androsterone (ADT) [41]. The activation of UGT2B15 and UGT2B17 genes would have a strong impact on the inactivation of androgens in LNCaP cells [41]. Overall, there appeared to be a concerted effort in preserving androgen in the tumor and may contribute to androgen-independence. Taken together, analysis of gene expression changes highlighted molecular mechanisms underlying the regulation of inflammatory responses and androgen response.

Our analysis also provides indirect evidence and mechanistic information to support LNCaP tumor xenograft progressed to a more androgen-independent phenotype than the parent LNCaP cells. For example, many genes involved in the modulation of the androgen receptor function were inhibited in the LNCaP xenograft tumor, including Hsp90AA2P, FLNA, and FKBP4. These proteins are all known to form complexes with the androgen receptor and enhance/modulate androgen receptor-mediated biological activities [71,72,73]. Downregulation of these proteins would suggest a deviation of the LNCaP tumor from androgen responsiveness of the parent LNCaP cells. Moreover, many signaling pathways that promote prostate cancer proliferation were upregulated. The genes listed as involved in androgen-independent pathway, such as MAPK12, and IGFBP7 (Supplementary Materials 1), all exhibited higher expressions in LNCaP xenograft tumor as compared to cultured cells. The activation of MAPK12 might be associated with the stress from the microenvironment [74] and IGFBP7 may be related to tumor angiogenesis [75]. Thus, upregulation of these genes may allow prostate cancer cells to bypass the dependence of proliferative activity on androgen and metastasize.

One of the pathways we focused on is related to chemokines and their receptors. We previously reported that CXCR7/AKCR3 may act as a decoy receptor for CXCL12 that counteract CXCR4-mediated LNCaP cell migration [28], a process that promote tumor metastasis. CXCL12 and CXCR7 were significantly altered in LNCaP xenograft tumor as compared to LNCaP cultured cells. CXCL12 was identified as the only chemokine changed in LNCaP xenograft tumor compared to cell culture (Table 4). The CXCL12 chemokine could be secreted by many types of cells, including immune cells such as primary blood monocytes [76], macrophages [77], as well as tumor cells from prostate and other tissues [78,79,80]. The activation of CXCL12 chemokine has been reported to increase the motility of LNCaP and PC3 cells [81] and induction of downstream pathways such as Akt-1 and metalloproteinases, which is involved in the regulation of prostate cancer cell migration [82]. CXCR7, the receptor for CXCL12, was significantly downregulated by −4.12-fold (Table 4). As we previously reported and in our current in vitro experiments, CXCR7 expression is downregulated by androgen and inflammation [28]. Given the overall downregulation of AR-mediated pathway in tumor, we consider the main regulator of CXCR7 in the xenograft tumor is inflammation. CXCL12′s regulation is a bit more puzzling as inhibitory effects were seen in inflammation and cell–matrix interaction in vitro model. It is unclear at this point what may trigger upregulation of CXCL12. Nonetheless, upregulation of CXCL12 would indicate attraction of immune cells and promotion of an overall proinflammatory environment. The downregulation of CXCR7, which would increase migration/metastatic potential of tumor cell, is lost due to the counterbalance of CXCR4. These results also suggest that CXCL12 may act as endocrine to further affect the tumor cell. These hypotheses, if proven correct, are important mechanisms contributing to prostate carcinogenesis and warrant further elucidation.

A recent study by Brady et al. 2020 [83] also addressed the gene transcriptional profiles in LNCaP 2D vs. 3D models but using whole-transcriptome microarray analysis, and RT-PCR. Consistent with our findings, the authors identified genes/pathways, associated with cell–cell interactions, and the extracellular matrix, enhanced in LNCaP xenograft tumor compared to cultured cells. Processes involved in translation and oxidative phosphorylation decreased in 3D compared to 2D. In our study using RNA-seq, CLC, and IPA analyses, we expand on the literature and provide additional information on genes/pathways associated with regulation of androgen, immune response, proliferation, metabolism, and hypoxic environment that are altered.

## 5. Conclusions

The ability to elucidate the effects of diet on prostate cancer and the potential mechanisms of action may depend on the experimental model used to test such effects. Therefore, it is important to have clear understanding of the molecular changes in the experimental model. In the present study, our analysis indicates extensive alteration of genes when LNCaP cells were injected into nude mice to form tumor xenograft. Bioinformatic analysis coupled with in vitro experimental models allows us to identify mechanisms by which genes and pathways may be regulated. The information highlights hypoxic condition and inflammation that may be the prominent mechanisms in this model. Moreover, these environmental factors may also contribute to androgen nonresponsiveness. These pathways are candidate pathways that may be modulated by diet or diet-derived compounds to provide protection against prostate cancer and point toward potential mechanisms. Overall, our data provide a valuable baseline for those who are interested in using cancer cell xenograft models to test diet/diet-derived compounds to elucidate mechanisms of action related to prostate carcinogenesis.

## Figures and Tables

**Table 1:** Upregulated and downregulated genes.

**A.** Upregulated genes			
**Symbol**	**Entrez Gene Name**	**Gene Symbol–Human (HUGO/HGNC/Entrez Gene)**	**Fold Change**
F3	coagulation factor III, tissue factor	F3	210.043
CREB3L1	cAMP responsive element binding protein 3 like 1	CREB3L1	182.705
ORM1	orosomucoid 1	ORM1	174.684
RGS2	regulator of G protein signaling 2	RGS2	138.156
TIMP1	TIMP metallopeptidase inhibitor 1	TIMP1	132.481
COL12A1	collagen type XII alpha 1 chain	COL12A1	110.37
MAGEB17	MAGE family member B17	MAGEB17	77.306
UNC80	unc-80 homolog, NALCN channel complex subunit	C2orf21	75.717
CA3	carbonic anhydrase 3	CA3	74.589
KRT75	keratin 75	KRT75	74.395
**B.** Downregulated genes			
**Symbol**	**Entrez Gene Name**	**Gene Symbol–Human (HUGO/HGNC/Entrez Gene)**	**Fold Change**
S100A7	S100 calcium binding protein A7	S100A7	−100.404
FCAMR	Fc fragment of IgA and IgM receptor	FCAMR	−62.565
UGT2B15	UDP glucuronosyltransferase family 2 member B15	UGT2B15	−62.323
UGT2B17	UDP glucuronosyltransferase family 2 member B17	UGT2B17	−54.141
GPX8	glutathione peroxidase 8 (putative)	GPX8	−35.5
UGT2B10	UDP glucuronosyltransferase family 2 member B10	UGT2B10	−35.444
TP53INP1	tumor protein p53 inducible nuclear protein 1	TP53INP1	−31.76
ID3	inhibitor of DNA binding 3, HLH protein	ID3	−21.13
UGT2B28	UDP glucuronosyltransferase family 2 member B28	UGT2B28	−20.846
HRNR	hornerin	HRNR	−20.03

**Table 2:** Top canonical pathways.

**A.** Top Pathways.				
**Ingenuity Canonical Pathways**	**−log (*p*-value)**	***p*-Value**	**Ratio**	**z-Score**
Aryl Hydrocarbon Receptor Signaling	4.79	0.0000162181	0.153	−1.155
AMPK Signaling	3.28	0.000524807	0.114	1
HOTAIR Regulatory Pathway	2.67	0.002137962	0.115	1.414
p53 Signaling	2.6	0.002511886	0.133	−0.577
Sperm Motility	2.57	0.002691535	0.104	0
Sirtuin Signaling Pathway	2.47	0.003388442	0.0954	−0.853
**B.** Top upregulated pathways				
**Ingenuity Canonical Pathways**	**−log (*p*-Value)**	***p*-Value**	**Ratio**	**z-Score**
AMPK Signaling	3.28	0.000524807	0.114	1
PTEN Signaling	1.7	0.019952623	0.103	1.387
HOTAIR Regulatory Pathway	2.67	0.002137962	0.115	1.414
LXR/RXR Activation	1.84	0.014454398	0.107	1.667
Protein Kinase A Signaling	1.74	0.018197009	0.0809	1.732
**C.** Top downregulated pathways				
**Ingenuity Canonical Pathways**	**−log (*p*-value)**	***p*-Value**	**Ratio**	**z-Score**
Cardiac Hypertrophy Signaling	1.75	0.017782794	0.0894	−2.683
Glioblastoma Multiforme Signaling	1.5	0.031622777	0.0926	−2.138
Acute Phase Response Signaling	1.47	0.033884416	0.0899	−2.53
Actin Nucleation by ARP–WASP Complex	1.44	0.036307805	0.114	−2.646
Cardiac Hypertrophy Signaling (Enhanced)	1.36	0.043651583	0.0737	−2.744
GNRH Signaling	1.35	0.044668359	0.0882	−2.309

**Table 3:** Top molecular and cellular functions changed.

Name	*p*-Value Range	Number of Molecules
Cellular Movement	2.37 × 10^−3^–1.00 × 10^−11^	194
Cellular Development	1.87 × 10^−3^–1.10 × 10^−9^	188
Cellular Growth and Proliferation	2.96 × 10^−3^–1.10 × 10^−9^	174
Cell Death and Survival	2.84 × 10^−3^–1.67 × 10^−7^	180
Cellular Assembly and Organization	1.16 × 10^−3^–7.16 × 10^−7^	119

**Table 4:** Alterations of chemokines and receptors in xenograft LNCaP tumor as compared to LNCaP cultured cells.

Gene_ID	Symbol	Expr Fold Change	*p*-Value
ENSG00000107562	CXCL12	26.703	9.61 × 10^−36^
ENSG00000144476	CXCR7	−4.1208	1.25 × 10^−6^

**Table 5:** Gene alteration associated with cell-matrix adhesion.

GENE_ID	Symbol	Entrez Gene Name	Fold Change	*p*-Value
ENSG00000164171	ITGA2	integrin subunit alpha 2	−6.122550677	6.29843 × 10^−20^
ENSG00000091409	ITGA6	integrin subunit alpha 6	−2.749811242	4.22149 × 10^−14^
ENSG00000150093	ITGB1	integrin subunit beta 1	−1.669372972	1.92104 × 10^−5^
ENSG00000090339	ICAM1	intercellular adhesion molecule 1	5.478055202	6.03337 × 10^−7^
ENSG00000105376	ICAM5	intercellular adhesion molecule 5	−5.772935224	7.54024 × 10^−8^

## Data Availability

The data presented in this study are available in article or Supplementary Materials.

## Supplementary Materials

The following are available online at https://www.mdpi.com/2072-6643/13/3/1000/s1, Figure S1: (A) AHR pathway. (B) HOTAIR pathway, Figure S2: IPA networks, Supplementary Materials 1: IPA gene list, Supplementary Materials 2: IPA top canonical pathway, Supplementary Materials 3: IPA networks, Supplementary Materials 4: IPA upstream regulator, Supplementary Materials 5: IPA causal networks.
