# Supplementary material for: Transcriptomic Analysis of LNCaP Tumor Xenograft to Elucidate the Components and Mechanisms Contributed by Tumor Environment as Targets for Dietary Prostate Cancer Prevention Studies
Source: Nutrients. 2021 Mar 19;13(3):1000. doi: 10.3390/nu13031000 (PMC8003580; doi:10.3390/nu13031000)
Supplement: Supplementary file 1 [file nutrients-13-01000-s001.zip › Lu's Nutrients Supplemental data/Supplemental Figures/Networks 1 through 25 (Figure S2) copy.docx]

IPA- NETWORKS #1-25

Network #1

Network 2

Network #3

Network #4

Network #5

Network #6

Network #7

Network #8

Network #9

Network #10


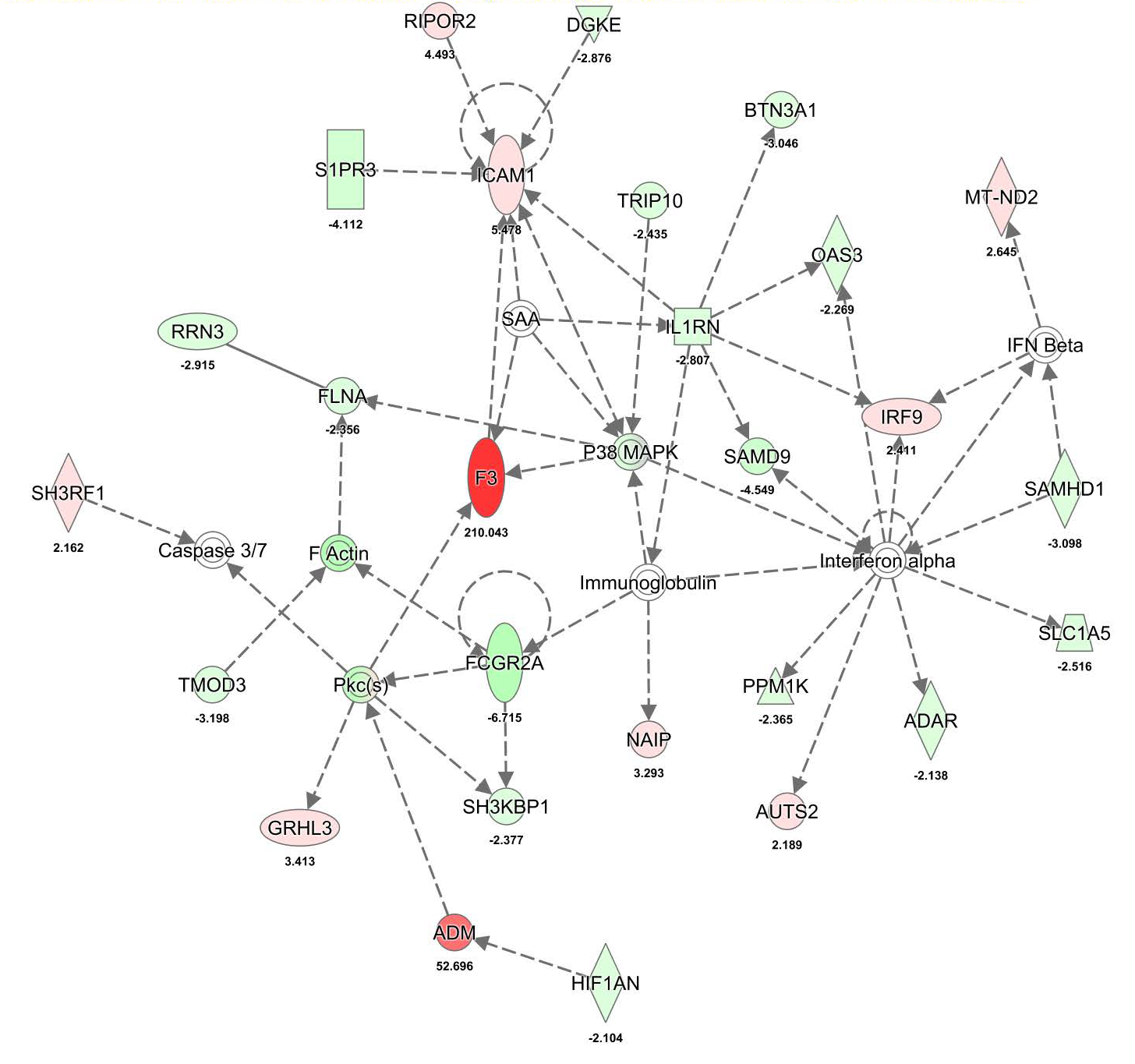


Network #11

Network #12

Network #13

Network #14

Network #15

Network #16

Network #17

Network #18

Network #19

Network #20

Network #21

Network #22

Network #23

Network #24

Network #25
