# Supplementary figures and images for: Transcriptomic Analysis of LNCaP Tumor Xenograft to Elucidate the Components and Mechanisms Contributed by Tumor Environment as Targets for Dietary Prostate Cancer Prevention Studies
Source: Nutrients. 2021 Mar 19;13(3):1000. doi: 10.3390/nu13031000 (PMC8003580; doi:10.3390/nu13031000)

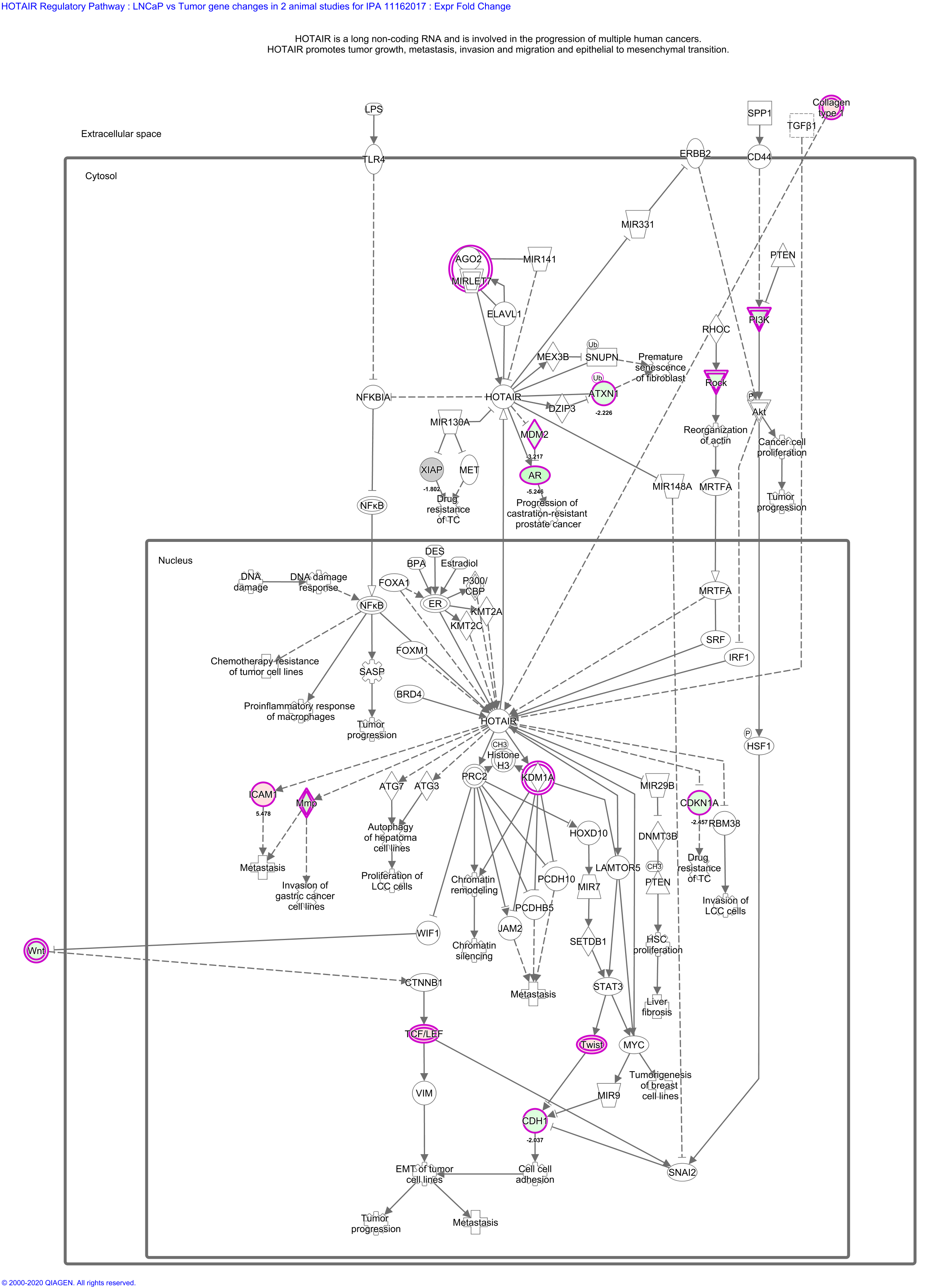

Supplement: Supplementary file 1 [file nutrients-13-01000-s001.zip › Lu's Nutrients Supplemental data/Supplemental Figures/HOTAIR canonical open pathway 2-7-2020 (Figure S1B) copy.tif]
